# Mitophagy in the Retinal Pigment Epithelium of Dry Age-Related Macular Degeneration Investigated in the *NFE2L2*/*PGC-1α*^-/-^ Mouse Model

**DOI:** 10.3390/ijms21061976

**Published:** 2020-03-13

**Authors:** Iswariyaraja Sridevi Gurubaran, Johanna Viiri, Ali Koskela, Juha M.T. Hyttinen, Jussi J. Paterno, Gréta Kis, Miklós Antal, Arto Urtti, Anu Kauppinen, Szabolcs Felszeghy, Kai Kaarniranta

**Affiliations:** 1 Department of Ophthalmology, Institute of Clinical Medicine, University of Eastern Finland, 70211 Kuopio, Finland; raja.sridevigurubaran@uef.fi (I.S.G.); johanna.viiri@uef.fi (J.V.); ali.koskela@uef.fi (A.K.); Jussi.Paterno@kuh.fi (J.J.P.); 2 Faculty of Medicine, Department of Anatomy, Histology and Embryology, University of Debrecen, Medical and Health Science Centre, Nagyerdei krt. 98, H-4032 Debrecen, Hungary; greta@anat.med.unideb.hu (G.K.); antal@anat.med.unideb.hu (M.A.); 3 Faculty of Health Sciences, School of Pharmacy, University of Eastern Finland, 70210 Kuopio, Finland; arto.urtti@uef.fi (A.U.); anu.kauppinen@uef.fi (A.K.); 4 Institute of Dentistry and Biomedicine, University of Eastern Finland, 70211 Kuopio, Finland; Szabolcs.Felszeghy@uef.fi; 5 Department of Ophthalmology and Kuopio University Hospital, University of Eastern Finland, 70029 Kuopio, Finland

**Keywords:** ageing, oxidative stress, mitochondrial damage, age-related macular disease, mitophagy, autolysosomal fusion, protein aggregates

## Abstract

Increased oxidative stress and mitochondrial damage are observed in protein aggregation diseases, such as age-related macular degeneration (AMD). We have recently reported elevated levels of oxidative stress markers, damaged mitochondria, accumulating lysosomal lipofuscin and extracellular drusen-like structures in the retinal pigment epithelial cells (RPE) of the dry AMD-resembling *NFE2L2*/*PGC1α* double knockout (dKO) mouse model. Here, we provide evidence of a disturbance in the autolysosomal machinery handling mitochondrial clearance in the RPE cells of one-year-old *NFE2L2*/*PGC1α*-deficient mice. Confocal immunohistochemical analysis revealed an upregulation of autophagosome marker microtubule-associated proteins 1A/1B light chain 3B (LC3B) as well as numerous mitophagy markers, such as PTE-induced putative kinase 1 (PINK1) and E3 ubiquitin ligase (PARKIN) together with damaged mitochondria. However, we detected no evidence of increased autolysosome formation in transmission electron micrographs or of colocalization of lysosomal marker LAMP2 (lysosome-associated membrane protein 2) and the mitochondrial marker ATP synthase β in confocal micrographs. Interestingly, we observed an upregulation of late autolysosomal fusion Ras-related protein (Rab7) in the perinuclear space of RPE cells together with autofluorescence aggregates. Our results reveal that there is at least a relative decrease of mitophagy in the RPE cells of *NFE2L2*/*PGC1α* dKO mice. This further supports the hypothesis that mitophagy is a putative therapy target in AMD-like pathology.

## 1. Introduction

Age-related macular degeneration (AMD) is the leading cause of blindness among the elderly [1,2,3]. AMD is a multifactorial disease which develops as a response to complex interactions between metabolic, genetic and environmental risks. The cellular pathology involves oxidative stress, protein aggregation and inflammation [4,5,6].

**Figure 1:**
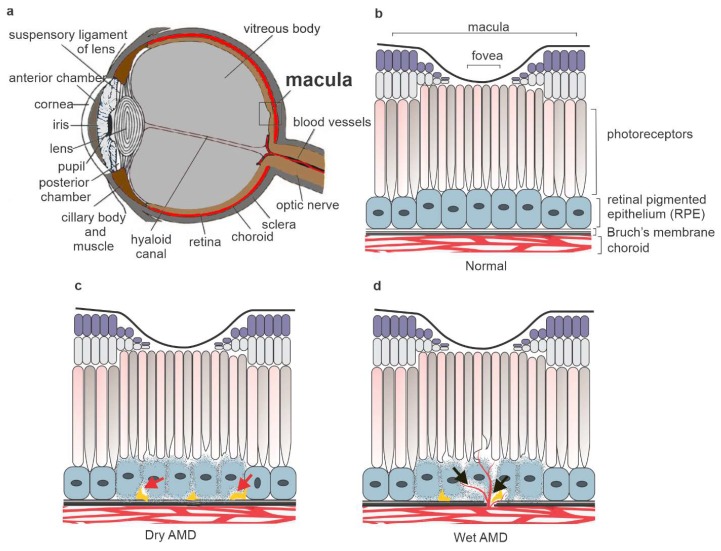
A cross-section of the human eye (**a**). The main outer retina layers in the healthy eye (**b**). The accumulation of drusen deposits (red arrow) and degeneration of retinal pigment epithelium (RPE) cells are detected in dry age-related macular degeneration (AMD) (**c**). Together with the dry AMD signs, choroidal neovascularization (black arrow) is a clinical hallmark of wet AMD (**d**).

A gradual loss of central vision due to degenerative and neovascular changes in the outer retina is characteristic of AMD (Figure 1). The early observed clinical hallmark is degeneration of the retinal pigment epithelium (RPE), involving the accumulation of protein, lipids and carbohydrates containing lysosomal lipofuscin and extracellular drusen deposits (Figure 1) [7,8,9,10]. Choroidal neovascularization is a diagnostical criterion for wet AMD. RPE cells have a key role in regulating the visual cycle and supporting the survival and maintenance of the functionality of photoreceptor cells (PRCs).

Functionally impaired mitochondria and cellular oxidation levels are linked to age-related neurodegenerative diseases, such as AMD [11,12,13,14]. RPE cells are known to be quiescent and their mitochondria are particularly prone to oxidative stress. In addition, each day RPE cells face enormous metabolic challenges in ensuring the diurnal endolysosomal clearance of lipid-rich photoreceptor outer segments (POS). Our recent observations support the hypothesis that oxidative stress and mitochondria damage are central factors in the pathology of AMD [15]. Once nuclear factor erythroid 2-related factor 2 (*NFE2L2*) and peroxisome proliferator-activated receptor gamma coactivator 1-alpha (*PGC-1α*) genes are silenced, it is possible to detect evidence of oxidative stress, protein aggregation and mitochondrial damage resembling the clinical signs of dry AMD [16,17]. 

In general, autophagy involves a bulky nonselective degradation pathway and selective form which maintains intracellular homeostasis by mediating and degrading cytoplasmic materials, such as misfolded proteins, damaged organelles, lipids and carbohydrates, by target-specific autolysosomal degradation. Subsequently, recycled materials are utilized for other cellular processes. In selective autophagy, specific cargos are ubiquitinated and recognized by autophagy adaptor proteins such as sequestosome-1 (p62/SQSTM1) preceding autolysosomal degradation [18,19,20,21]. Selective autophagy can be classified according to the specific target, e.g., mitophagy (mitochondria), lipophagy (lipid droplets), glycophagy (glycogen) and ribophagy (ribosomes) [22,23]. 

Unlike canonical selective autophagy, once mitochondrial damage and depolarization has occurred, the serine/threonine kinase PINK1 (PTEN-induced putative kinase 1) serves as a sensor for the mitochondrial polarization state. Under normal conditions, the polarized mitochondrial PINK1 is imported into the mitochondria and becomes rapidly degraded by combined activity of protease PARL (presenilin associated rhomboid-like protein) and proteasomes [24,25]. Mitochondrial depolarization results in the accumulation of PINK1 on the outer mitochondrial membrane (OMM) and recruitment of PARKIN (465-residue E3 ubiquitin ligase) from the cytosol. PINK1 at the OMM phosphorylates ubiquitin at serine 65 [26,27,28] and the ubiquitin-like domain of PARKIN at serine 65 [29]. Once recruited and activated, PARKIN E3 ligase activity ubiquitinates numerous downstream autophagosome-related proteins and stimulates the local formation of autophagosomes with LC3 (microtubule-associated proteins 1A/1B light chain 3B) via their LIR motifs (LC3 interacting region) [30,31]. Once the autophagosome has matured, the RAS-related GTP-binding proteins (Rab family proteins) become involved in the modulation of lysosome and autophagosome fusion process and the subsequent cargo degradation by hydrolytic enzymes. In particular, Rab5 is known to associate with the autophagosomal precursor, whereas Rab7 is involved in the late endolysosomal process [32,33].

In this study, we show that absence of *NFE2L2*/*PGC-1α*, the master regulators of antioxidant production and mitochondrial biogenesis, leads to disturbed mitophagy in the mouse RPE cells. The data suggest that there is a functional initiation of the mitophagy, but it fails during the late stages of process. 

## 2. Results

### 2.1. Mitochondria Validation 

**Figure 2:**
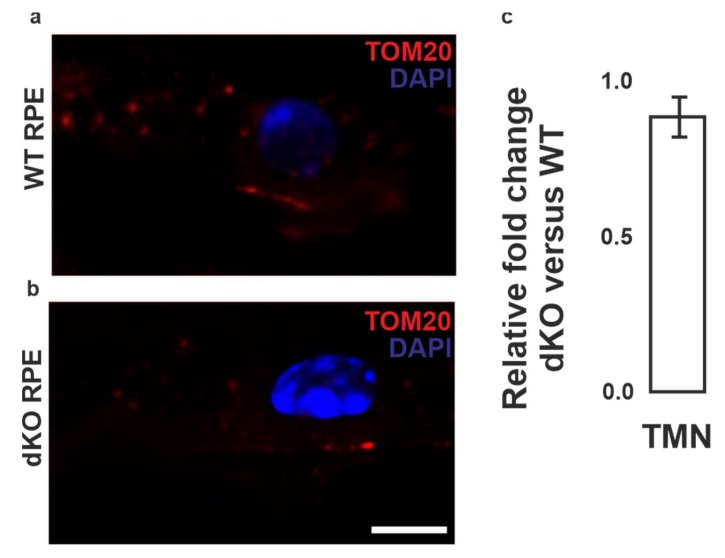
Confocal microscopy analysis of mitochondria in RPE cells. Mitochondrial marker TOM20 (red) binds to OMM of both healthy and damaged mitochondria in RPE cells of wild type (WT) (**a**) and double knockout (dKO) mice (**b**). The relative changes of the total mitochondrial number in dKO versus WT are shown, n = 5 images per eye/animal (**c**). Scale = 5 µm. TMN, total mitochondrial number.

Initially we examined the condition of the PRCs, Bruch’s membrane and choroid of one-year-old wild type (WT) and double knockout (dKO) mice (Supplementary Figure S1). The WT mice displayed the normal phenotype of PRCs, RPE and intact Bruch’s membrane in retina. The dKO animals [15] were characterized with typical AMD signs, such as an accumulation of drusen-like deposits. The total number of mitochondria in WT and dKO was analyzed using mitochondrial import receptor unit TOM20 immunostaining (Figure 2). We observed no statistical TOM20 staining difference between WT and dKO in mitochondria. Since TOM20 recognizes both healthy and damaged mitochondria, we conducted ultrastructural transmission electron microscope (TEM) analysis. A decline in viable mitochondria and higher mitochondrial damage were detected in dKO RPE cells (Supplementary Figure S1).

### 2.2. PINK1/PARKIN in Primary Mitochondrial Quality Control System

**Figure 3:**
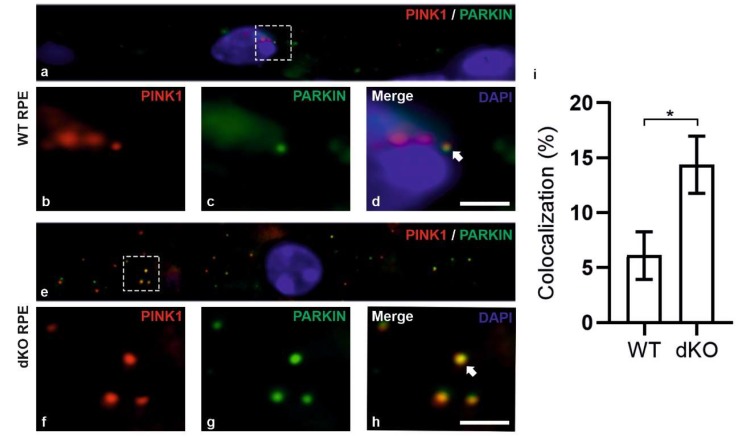
Confocal microscopy analysis of the mitophagy initiation in the RPE cells by staining PINK1 and PARKIN. One-year-old WT and dKO mice focusing on the RPE cells in the vicinity of the optic nerve (**a**,**e**). PINK1 (**b**, red) and PARKIN (**c**, green) were double-stained and the merged image (**d**) was used to count the colocalized puncta from WT. Similarly, in dKO PINK1 (**f**, red) and PARKIN (**g**, green) were double-stained, and the merged image (**h**) was used to count the colocalized puncta. In dKO, we observed a ~7% decrease in the total number of puncta; however, the number of colocalizations was increased by ~118% (**i**). Scale = 5 µm. *p = 0.01.

**Figure 4:**
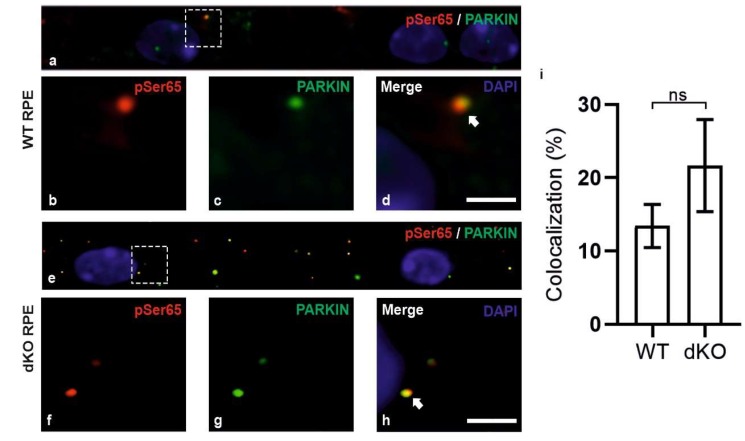
WT and dKO RPE cells (**a**,**e**) were double-stained for pS65 (**b**,**f**) and PARKIN (**c**,**g**) and analyzed by confocal microscopy. In dKO, we observed a ~33% increase in the total puncta with a ~115% increase in colocalization as compared to WT (**d**,**h**,**i**). Scale = 5 µm. p = 0.2. ns—Nonsignificant.

**Figure A1:**
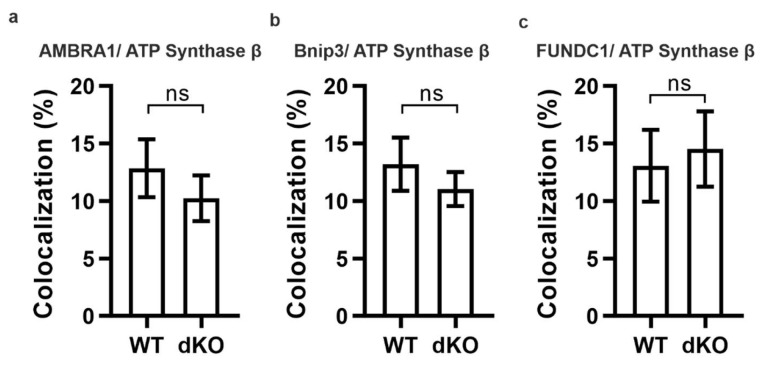
Representative graphical data from double-staining of AMBRA1 (**a**, p = 0.4), Bnip3 (**b**, p = 0.4) and FUNDC1 (**c**, p = 0.7) with ATP synthase β. There was no statistically significant difference in the colocalization analysis between one-year-old WT and dKO. ns—Nonsignificant.

During mitophagy, PINK1 is known to be stabilized on the OMM and recruit PARKIN along with the series of other autophagy-related proteins to initiate local autophagosome formation. The initiation of mitophagy process in RPE cells of dKO was initially assessed by analyzing the recruitment of mitochondrial sensor PINK1 and PARKIN on the damaged mitochondria using double staining immunofluorescence (Figure 3). In dKO, the RPE cells revealed a ~7% reduction in total puncta. However, the colocalization was significantly increased by ~118% as compared to WT (Figure 3i). The occurrence of colocalization was observed in ~6% of WT and in ~12% of dKO, as normalized to the total number of puncta from each channel. Next, PINK1/PARKIN colocalization with phospho-ubiquitin serine 65 (pS65) was assessed (Figure 4). PARKIN phosphorylated at the Ser65 by PINK1 is known to specifically bind to pS65 [34,35]. We observed a ~33% increase in the total puncta in dKO along with an increased colocalization of pS65 and PARKIN by 115% in dKO RPE cells (Figure 4i). The increase in colocalization of pS65/PARKIN reflected the recruitment of phosphorylated PARKIN to the damaged mitochondria and the initiation of mitophagy. Studies have also shown PINK1/PARKIN-unrelated mitophagy receptors that may contribute to the mitophagy activation [20]. We studied the role of oxidative stress and hypoxia-related ubiquitin-dependent and ubiquitin-independent mitophagy receptors in dKO mice model. However, AMBRA1 (autophagy and beclin-1 regulator 1), Bnip3 (BCL2 interacting protein 3) and FUNDC1 (FUN14 domain-containing 1) did not colocalize with mitochondrial marker ATP synthase β, indicating that these receptors do not actively participate in mitophagy initiation in this model (Appendix A, Figure A1). In summary, our results suggest that PINK1/PARKIN control the regulation of mitophagy in situations of oxidative stress and mitochondrial damage [36].

### 2.3. Relative Decrease in Autolysosomal Clearance and Impaired Mitophagy Process

**Figure 5:**
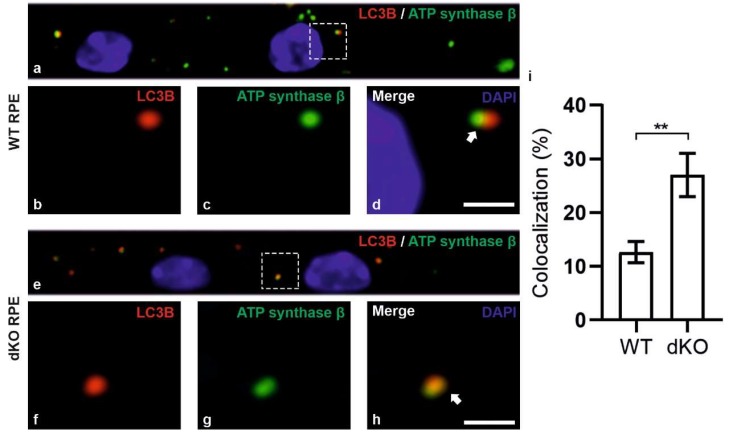
Confocal microscopy analysis of autophagosome marker LC3B and mitochondrial marker ATP synthase β in WT and dKO RPE cells (**a**,**e**). The colocalization of LC3B (**b**,**f**) and ATP synthase β (**c**,**g**) will show the amount of local autophagosome formation. Our image analysis showed a ~158% increase in the LC3B and ATP synthase β colocalization in dKO compared to in WT (**d**,**h**,**i**). Scale = 5 µm. **p < 0.0025.

After PINK1/PARKIN have been localized in the outer membranes of damaged mitochondria, then autophagosome should be formed. We analyzed the formation of the autophagosome by undertaking double staining of LC3B and mitochondrial marker ATP synthase β (Figure 5). In principal, once mitophagy has been activated, the proteins form mature autophagosomes before fusing with a lysosome. The colocalization of LC3B and ATP synthase β (Figure 5d,h) are evidence of the formation of the autophagosome. In dKO RPE cells, we observed a statistically significant ~158% increase in the positive colocalization, preceding a ~21% increase in total number of puncta as compared to WT cells (Figure 5i). In addition, the correlation of colocalization within WT was ~12%; in dKO it was ~21%. These data reveals the relative increase in autophagosome formation, particularly the form mediated via PINK1/PARKIN pathway. 

**Figure 6:**
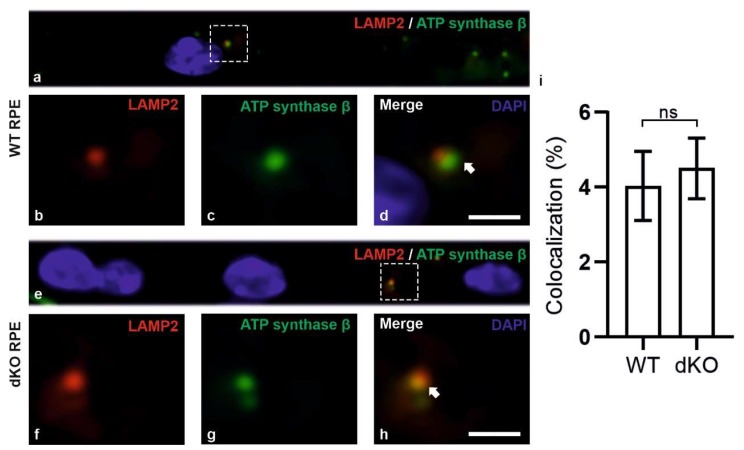
Confocal microscopy analysis of lysosomal marker LAMP2 and mitochondrial marker ATP synthase β in WT and dKO RPE cells (**a**,**e**). The colocalization of LAMP2 (**b**,**f**) and ATP synthase β (**c**,**g**) shows the amount of autolysosome formation. Image analysis showed no significant changes in LAMP2 and ATP synthase β colocalization in dKO animals compared to WT (**d**,**h**,**i**). Scale = 5 µm. p = 0.70. ns—Nonsignificant.

In summary, we have observed that damaged mitochondria are marked by PINK1/PARKIN and that autophagosomes with mitochondrial cargo are formed more extensively in the dKO mice than in the corresponding WT animals. Subsequently, in an attempt to track the clearance of the cargo, we applied the double-staining of the lysosomal marker LAMP2 and mitochondrial marker ATP synthase β (Figure 6). In dKO RPE cells, the total number of puncta and the degree of colocalization were increased only by ~8% and ~20%, respectively. This clearly indicates that there is a profound 8-fold decrease in the numbers of autolysosomes (Figure 6i) as compared to autophagosomes (Figure 5i). Hence, one can conclude that, in conditions of oxidative stress and mitochondrial damage, the process of mitophagy could be impaired in our *NFE2L2*/*PGC-1α* dKO mice model, which has many of the characteristics of AMD. 

**Figure 7:**
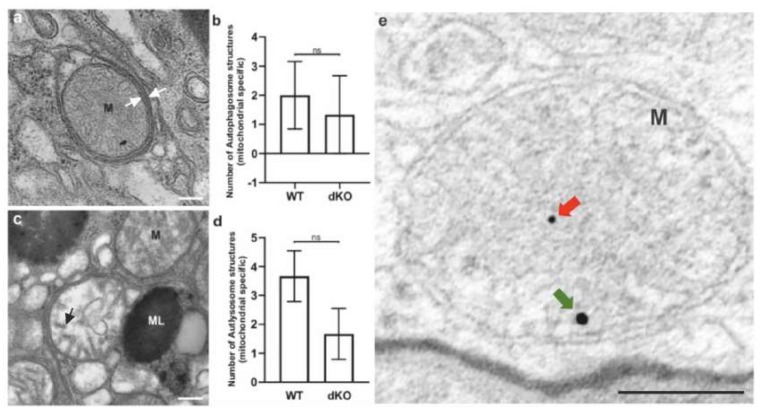
Representative TEM pictures of autophagosome and autolysosome of selective autophagy, i.e., mitophagy. Mitochondria (M) fully enveloped within an autophagosome (**a**). The white arrows indicate the double membrane of matured autophagosome. The amount of mitochondria-specific early autophagosomes between WT and dKO was statistically (**b**) nonsignificant (ns). Highly degraded mitochondria, representing a late autolysosome (the black arrow shows degrading cristae) (**c**). No statistical significance was found in the number of the late autolysosome structures between WT and dKO (**d**). The red and green arrows indicate PINK and PARKIN immunogold particles in the dKO RPE samples, respectively (**e**). ML, melanosome. Scale = 0.5 µm (a and c), scale = 0.2 µm (e). ns—Nonsignificant.

Since the staining of LAMP2/ATP synthase β did not show significantly increased colocalization, we used transmission electron microscopy (TEM) and immunogold labeling of PINK and PARKIN to detect mitochondria-specific autophagic organelles. We were unable to identify a clear difference in the number of mitochondria-specific autophagosomes and reduced formation of autolysosomes when comparing between WT and dKOs, although, the latter animals had revealed a decreased tendency in line with our colocalization results (Figure 7). Moreover, no PINK or PARKIN immunogold signals were seen in WT, while dKO samples showed positivity inside the mitochondria (Figure 7e).

### 2.4. Aggregates Colocalize with Rab7

**Figure 8:**
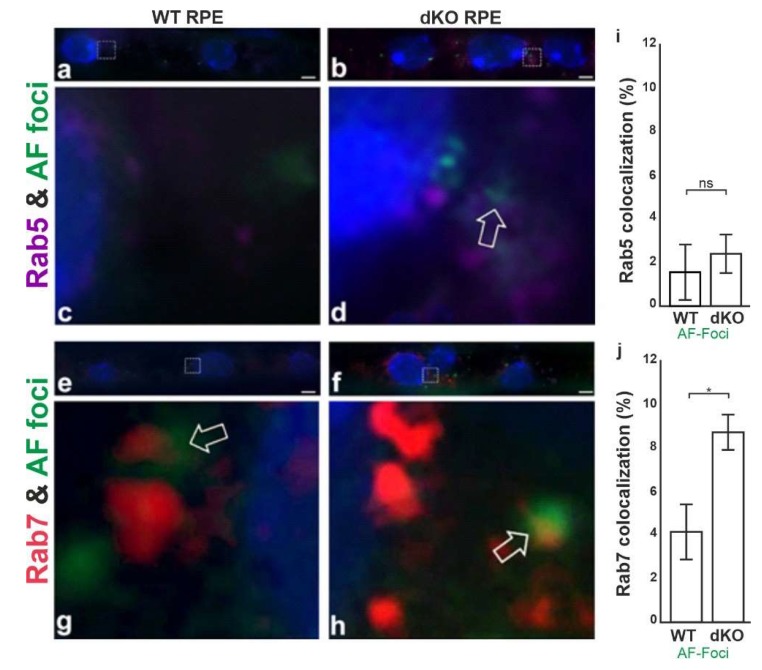
Representative high-power confocal microscopy images of WT (**a**,**c**,**e**,**g**) and dKO (**b**,**d**,**f**,**h**) show that Rab5 (**a**–**d**) and Rab7 (**e**,**f**) proteins are present throughout the cytoplasm of RPE cells but are prominently concentrated in the perinuclear space. However, they were differently expressed in WT and dKO RPE cells. Note that the formation of autofluorescence spots (AF) was more prominent in dKO samples (**c**,**g**), whereas WT RPE (**a**,**f**) exhibited only a few AF foci. The confocal plane and pinhole settings for the squared regions of WT and dKO were chosen to reveal mainly details of the pericellular space for Rab5, Rab7 and the distribution of AFs spots in WT and dKO samples, where the white arrow indicates the areas of possible colocalization. Quantification of Rab5 (**i**) and Rab7 (**j**) with AF foci colocalization. Colocalization analysis revealed a low association of early endosomal marker Rab5 with AF-foci in both genotypes studied, whereas the colocalization of the late endosomal marker Rab7 with AF-foci was clearly increased in dKO RPE compared to WT samples, mean ± SD. Scale = 1 μm. *p < 0.05. ns—Nonsignificant.

Lipofuscin are yellowish autofluorescence pigments that accumulate as a result of free-radical damaged proteins and lipids during ageing [37]. Since our model displays relatively reduced mitophagy, we were interested to determine whether the autophagosome and lysosomal fusion process might be disrupted. Ras-related proteins Rab5 and Rab7, small GTPase proteins and regulators of the early and mature autophagosome formation were analyzed with respective antibodies [38,39]. Confocal microscopy analysis showed that the green autofluorescence (AF) structures in WT and dKO RPE cells only partially co-stained for Rab5 (Figure 8). However, a clear co-staining between AF and Rab7 was detected in the perinuclear space of dKO samples (Figure 8d,h). The AF and early endosome marker Rab5 also showed some degree of colocalization in the dKO RPE cells (Figure 8c,g). Moreover, the dispersed cytoplasmic AF foci in both genotypes seemed to be smaller than those present in the perinuclear aggregates in WT (Figure 8c,d) and dKO (Figure 8g,h). AF represent a heterogenous signal in number and intensity within RPEs, and a higher number of aggregates were detected in dKO RPE cells as compared to WT samples (Figure 8i). 

As far we are aware, this is the first study that has demonstrated a deficiency of mitophagy in the *NFE2L2*/*PGC-1α* knockout mice. There is evidence that RPE cells attempt to initiate mitophagy, but autophagy flux to ensure mitochondrial clearance does seem to be disturbed. 

## 3. Discussion 

During the aging process, increased oxidative stress leads to a dysfunction of mitochondria and detrimental protein aggregation [37,40,41,42,43]. Dysfunctional mitochondria and the elevated generation of ROS are among the earliest events occurring in the progression of the numerous neurodegenerative protein aggregation diseases, such as Alzheimer’s and Parkinson’s diseases [44,45]. Since it is essential that cells have healthy mitochondria for energy production as well as for several cytoprotective cellular processes, dysfunction in these processes would be predicted to exert profound harmful consequences in AMD. Mitochondria undergo constant fission and fusion in order to meet the demands of the cells [46]. In conditions of stress, both mitochondrial fission and fusion work in parallel to maintain the function of these organelles. An increase in mitochondrial fission leads to mitochondrial fragmentation, whereas fusion results in mitochondrial elongation. Failures in the components of this machinery lead to cellular degeneration and even cell death [47]. We exploited our *NFE2L2*/*PGC-1α* dKO mice model to gain detailed insights into mitophagy during conditions of oxidative stress and damaged mitochondria.

The functional role of PINK1/PARKIN in mitophagy has been extensively reviewed elsewhere [48,49,50,51]. Here, we focused on the clearance capacity of mitophagy alone. Our immunohistochemical colocalization studies in RPE cells of *NFE2L2*/*PGC-1α* dKO displayed a significant increase in the PINK1/PARKIN levels. The results were in line with previous observations in the PINK1-deleted mammalian model [52], *Drosophila* PARKIN mutants [53], and induced mitochondrial damage in HeLa [54] and ARPE-19 cell lines [55]. The levels of PINK1/PARKIN were accompanied by equal pS65/PARKIN levels. Hence, our data clearly fit with the perception of oxidative stress and damaged mitochondria in *NFE2L2*/*PGC-1α* dKO. The immunoelectron microscopy analysis showed evidence of a strong colabelling of PINK1 and PARKIN in dKOs. No, or only a few, PINK1/PARKIN signals were detected in WT animals. These findings indicate elevated mitophagy initiation in dKO mice.

**Figure 9:**
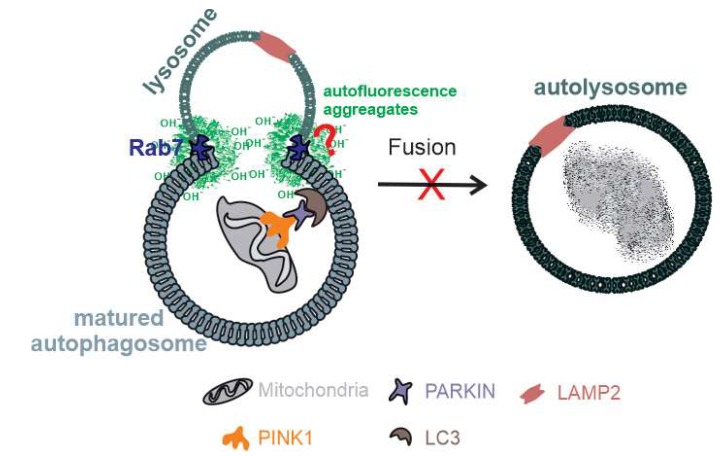
A schematic representation of disturbed selective autophagy in RPE cells of *NFE2L2*/*PGC-1α* dKO. Under conditions of chronic oxidative stress and mitochondrial damage, there is evidence of incomplete mitophagy in our model due to the accumulation of reactive autofluorescence aggregate linked Rab7 colocalization. However, more studies will be required to clarify the association between Rab7, an autophagosome-related vesicular transport protein, and oxidized cross-linked materials.

The ubiquitinated mitochondria are recognized by LC3B either directly or via adopter proteins and become sequestered into autophagosomes [56,57,58]. High levels of LC3 and ATP synthase β colocalization were detected in the dKO samples. This suggests that there is a buildup of critically demanding sections of the mitophagy (autophagosome) process in RPE cells if they lack the *NFE2L2*/*PGC-1α* genes. Interestingly, we did not observe an increase in the late-stage markers of mitochondrial clearance, LAMP2/ATP synthase β colocalization. Therefore, we examined the autophagosome and lysosome fusion process. The late autophagosomal marker Rab7 colocalized with autofluorescence protein aggregate components, especially in the perinuclear space of RPE cells in dKOs. This observation seems to indicate that there is a dysfunction in the autolysosomes due to the influence of detrimental oxidized cross-linked proteins in the lysosomal enzymes [59]. Naive RPE cells exposed to oxidative compounds can effectively maintain functional mitochondrial–lysosomal axis and phagocytic POS clearance [60]. During aging and in pathological states, this capacity declines, disturbing the function of autophagy and interfering with the ubiquitin proteolytic system [61]. We detected increased staining of both Rab5 and Rab7 together with perinuclear autofluorescence deposits. Lipofuscin has been reported to inhibit lysosomal protein degradation and may cause AMD [62]. However, more experiments will be needed to clarify the relationship between Rab7 and the presence of highly reactive autofluorescence aggregates [63]. Our results suggest that if *NFE2L2*/*PGC-1α* signaling is disturbed, then the accumulation of oxidized undigested aggregates, including lipofuscin, can potentially hinder the critical mitophagy process in RPE cells (Figure 9). 

Our study highlights the importance of autolysosomal capacity and its components during chronic oxidative stress and mitochondrial damage. There is an increasing body of evidence revealing the importance of autophagy in the regulation of mitochondrial homeostasis. Therefore, mitophagy may represent a novel therapy target in the prevention or treatment of dry AMD.

## 4. Materials and Methods

### 4.1. Ethics and Animal Experiments

All the experimental protocols were conducted in accordance with the institutional guidelines of Animal Ethics Committee of the Provincial Government of Southern Finland and with the guidelines of European Community Council Directives 86/609/EEC. We have gained permission for the use of animals in research (ESAVI/8893/04.10.07/2014). According to our recent documentation, dry AMD-like *NFE2L2*/*PGC-1α* dKO [15] 1-year-old male mice (n = 3, six eyes) and sex- and age-matched wild-type controls (n = 3, six eyes) were used in the study. All animals were bred and housed in the Laboratory Animal Centre of University of Eastern Finland, Kuopio. The 3R-principles were implemented in the animal studies. 

### 4.2. Genotyping and Tissue Preparation 

The global double knockout (dKO) mice were made by knocking down *PGC-1α* and *NFE2L2*. The animals were genotyped using *PGC-1α* with four-primer PCR. The primer sequences were: WTA, 5′-CCA GTT TCT TCA TTG GTG TG; WTB, 5′-ACC TGT CTT TGC CTA TGA TTC; KOA, 5′-TCC AGT AGG CAG AGA TTT ATG AC; KOB, 5′-CCA ACT GTC TAT AAT TCC AGT TC. The following *NFE2L2* genotypes were studied with three primers: LacZ, 5′-GCG GAT TGA CCG TAA TGG CAT AGG; *NFE2L2*-5′, 5′-TGG ACG GGA CTA TTG AAG GCT G; *NFE2L2*-3′, GCC GCC TTT TCA GTA GAT GGA CG [15]. 

Both reactions included 4 μL of DNA extracted from mouse ears, reaction buffer, 100 μM of each dNTPs, 1.5 mM of MgCl_2_, 1 μM of each primer and 1.2 U of DreamTaq DNA polymerase (Thermo Fisher Scientific Waltham, MA USA), in a reaction volume of 30 μL. For the PGC-1α, samples were denatured at 95 °C for 5 min, followed by 39 cycles at 95 °C for 30 s, 58 °C for 30 s, 72 °C for 30 s, and a final extension at 72 °C for 7 min. For the *NFE2L2* genotyping, the reaction conditions were denaturation at 95 °C for 5 min, followed by 35 cycles at 95 °C for 30 s, at 59 °C for 30 s, at 72 °C for 45 s, and a final extension at 72 °C for 7 min. The amplicon sizes for the wild-type and KO alleles of PGC-1α were 600 and 400 bp, respectively; the WT and KO alleles for *NFE2L2* were 700 and 400 bp, respectively (Supplementary Figure S2). 

In the tissue preparation, the animals were sacrificed with cervical dislocation and the eyes were immediately carefully enucleated and placed in PBS (pH 7.4), followed by fixation in 4% paraformaldehyde in 0.1 M phosphate buffer for 24–48 h and ethanol dehydration. Five-micrometer-thick parasagittal serial sections were cut from embedded blocks with a microtome (SM2000 R, Leica, Heidelberg, Germany) for immunohistochemical analysis. 

### 4.3. Immunohistochemical Staining

Tissues sections were deparaffinized using xylene and rehydrated. Then, the glass sections were incubated for 25 min in the dark with 0.5% Sudan black B (Acros Organics, USA) in 70% EtOH. The washed slides were pretreated with Tris-based antigen unmasking solution (Vector Laboratories Inc., CA) for 7.5 min at 90 °C. In the analysis of autofluorescence with Rab5 and Rab7 (Table 1), the slides were pretreated with Tris- and Citrate-based antigen unmasking solutions (Vector Laboratories Inc., CA), respectively, for 5 min at 90 °C. The sections were encircled with a PAP pen and quenched with 0.1 M glycine in PBS for 10 min prior to a 0.1% Triton-X wash for 10 min before continuing with blocking for 30 min. Quenched slides were incubated with 20% goat serum for 30 min before adding the first primary antibodies (Table 1) and incubated overnight at 4 °C. The sections were incubated at room temperature for 30 min and washed in the dark for 10 min. Then, the first secondary antibodies were added and incubated for 3 h, followed by a second primary antibody incubation for 3 h. Finally, DAPI (Sigma Aldrich, USA) was added at a ratio of 1:10,000 and incubated for 30 min followed by a 5-min wash with TBS. Then, the slides were mounted using Mowiol mounting media and stored in the dark at room temperature. 

The secondary antibodies were goat anti-rabbit Alexa Fluor 488 and 594 (A11034/ A11037) and goat anti-mouse Alexa Fluor 488 and 594 (A11029/ A11032) (ThermoFisher Scientific, USA) diluted at 1:500. 

### 4.4. Confocal Imaging

The stained sections were examined with a confocal microscope (Zeiss AX10 Imager A2, Zeiss, DE) using a 63× (NA:1.42. Plan Apochromat) oil (Zeiss Immersol™, DE) immersion objective. The microscopic settings were kept identical for all pictures taken and held constant during imaging. Representative high-power microphotos were taken close to the vicinity of the optic nerve with ZEN blue v2.3 (Carl Zeiss Microscopy, DE). At least nine repetitive images were taken from each section for all markers. Images were color enhanced using Adobe photoshop for visual representation. 

### 4.5. Colocalization Analysis

All the captured images were converted into 8-bit and processed using ImageJ v1.52a (https://imagej.nih.gov/ij/). The background was subtracted using a default rolling ball radius method. Regions of interest (ROI) were drawn over RPE cell layer followed by colocalization analysis using 2 channel spots colocalization analyzer ComDet v0.3.7 (https://imagej.net/Spots_colocalization_(ComDet)), an ImageJ plugin. All the colocalization analysis were blind quantified at least by three independent researchers. The total number of puncta from each color (channel) was calculated and the corresponding correlations of colocalization were measured. The degree of colocalization between WT and dKO was analyzed after normalization of the total number of puncta. 

### 4.6. Transmission Electron Microscopy (TEM) Preparation and Mitochondria-Specific Autophagosome and Autolysosome Analysis

Samples were processed and analyzed according to our recent publication [15]. Briefly, the specimens were washed in 0.1 M cacodylate buffer containing 3.7% (w/v) saccharose and sequentially dehydrated in 30% (v/v) ethanol at 4 °C, 50% (v/v) ethanol at 0 °C and 70% (v/v) ethanol at −20 °C. The specimens were then immersed in a 1:1 mixture of 70% ethanol and medium grade LX112 resin (Ladd Research Industries, USA) at 4 °C with several changes of pure resin at 4 °C and room temperature and finally polymerized in gelatin capsules for 1 day at 45 °C. One-micron semithin sections were cut with a Reichert Ultracut E microtome (Leica Microsystems Inc, IL, USA), stained with 1% toluidine blue and examined with a light microscope to find the localization of interest (RPE) prior to further TEM sectioning. Ultrathin sections were cut with a microtome (Ultracut; Leica, Bensheim, Germany) and mounted on uncoated nickel grids. Mitochondria-specific autophagosome and autolysosome were assessed using JEM-1010 TEM (Tokyo, Japan) transmission electron microscope. Similarly, as conducted with the histological samples, representative areas per sample were selected by collecting RPEs from each of the individual one-year-old WT and dKO samples which were close to the vicinity of the optic nerve. In the subsequent analysis, the cells in the region of interest were randomly selected (n = 18). The morphological criteria for healthy and damaged mitochondria selection are shown in Supplementary Figure S2.

### 4.7. Postembedded Immunolabelling 

In the postembedding immunogold labeling, tissue specimens (approximately 5 × 5 mm in size) were fixed immediately after death in freshly prepared 3% (w/v) paraformaldehyde and 0.1% (v/v) glutaraldehyde in 0.1 M cacodylate buffer (pH 7.4), and the eyeballs were processed according to our previous publication [15]. Briefly, the specimens were washed in 0.1 M cacodylate buffer containing 3.7% (w/v) saccharose and sequentially dehydrated in 30% (v/v) ethanol at 4 °C, 50% (v/v) ethanol at 0 °C and 70% (v/v) ethanol at −20 °C. The specimens were then immersed with a 1:1 mixture of 70% ethanol and medium grade LX112 resin (Ladd Research Industries, USA) at 4 °C with several changes of pure resin at 4 °C and room temperature and finally polymerized in gelatin capsules for 1 day at 45 °C. One-micron semithin sections were cut with a Reichert Ultracut E microtome (Leica Microsystems Inc, IL, USA), stained with 1% toluidine blue and examined with a light microscope to find the localization of interest (RPE) for further TEM sectioning. Ultrathin sections were cut with a microtome (Ultracut; Leica, Bensheim, Germany) and mounted on uncoated nickel grids.

The method of postembedding immunogold labeling was based on previous reports [64,65]. The mouse monoclonal antibody PARKIN (sc-32282, Santa Cruz Biologicals, Dallas, TX, USA) and rabbit polyclonal PINK1 (BC100-494, Novus Biologicals, Briarwood Avenue, Building IV Centennial, CO, USA) antibodies were applied to detect respective epitopes. Appropriate secondary antibodies conjugated to 10 nm (PINK1) and 15 nm (PARKIN) colloidal gold particles (diluted 1:30; Bio Cell, Cardiff, Wales, UK) were used to detect the primary antibodies. Briefly, in the postembedding immunogold labeling, ultrathin sections of WT and dKO samples were incubated for 90 min successively in drops of the mixture of these antibodies followed by a mixture of gold-conjugated goat anti-mouse (15 nm) and gold-conjugated goat anti-rabbit (10 nm) secondary antibodies for 2 h [66]. After several washing steps, grids were briefly stained with uranyl acetate and lead citrate and examined with a transmission electron microscope (JEM-1010 TEM, Jeol, Tokyo, Japan). In the quantification of PINK and PARKIN immunogold particles in RPE from WT and dKO animals, a total of 40 mitochondria from each group were analyzed, and mitochondria with double PINK1 (10 nm) and PARKIN (15 nm) immunogold labeling were identified.

### 4.8. Statistics

All data are presented as mean ± SEM (standard error of the mean). A two-tailed, unpaired Student’s *t*-test was used to determine the statistical significance in the double-staining colocalization study as well as in signal intensity analysis. P < 0.05 was considered statistically significant; “ns” represents statistical nonsignificance.

## Figures and Tables

**Table 1:** List of primary antibodies.

Primary Antibodies Against	Isotope	Working Dilution	Supplier/Catalogue Number
ATP synthase β	Monoclonal	1:100	TF-A-21351
AMBRA1	Polyclonal	1:100	NBP2-32640
Bnip3	Polyclonal	1:200	NBP1-77683
FUNDC1	Polyclonal	1:200	NBP1-81063
HSP75 (TRAP1)	Monoclonal	1:100	AB2721
LAMP2	Polyclonal	1:500	NB300-591
LC3B	Monoclonal	1:100	CT-3868
PARKIN	Monoclonal	1:100	NBP2-29838
Phospho-ubiquitin-Ser65	Polyclonal	1:100	ABS1513-I
PINK1	Polyclonal	1:250	BC-100-494
Rab5	Monoclonal	1:50	SC-46692
Rab7	Monoclonal	1:100	AB-50533
TOM20	Monoclonal	1:100	SC-17764

## Supplementary Materials

Supplementary materials can be found at https://www.mdpi.com/1422-0067/21/6/1976/s1.

## Abbreviations

AMD	Age-related macular degeneration
RPE	Retinal pigment epithelial cells
dKO	Double knockout
WT	Wild type
*NFE2L2*	Nuclear Factor, Erythroid 2 Like 2
*PGC1α*	Peroxisome proliferator-activated receptor gamma coactivator 1-alpha
LC3B	Microtubule-associated proteins 1A/1B light chain 3B
PINK1	PTEN-induced kinase 1
PARKIN	E3 ubiquitin ligase
LAMP2	Lysosome-associated membrane protein 2
Rab(5/7)	RAS-related GTP-binding proteins (5/7)
PRCs	Photoreceptor cells
POS	Photoreceptor outer segments
p62/SQSTM1	Sequestosome-1/ ubiquitin-binding protein p62
OMM	Outer mitochondrial membrane
NBR1	Neighbor of BRCA1 gene 1 protein
OPTN	Optineurin
AMBRA1	Autophagy and beclin-1 regulator 1
LIR	LC3-interacting region
Bnip3	BCL2 interacting protein 3
Nix/Bnip3L	BCL2-like 3
FUNDC1	FUN14 domain-containing 1
Bcl2L13	BCL2-like 13
TOM20	Mitochondrial import receptor subunit TOM20
TEM	Transmission electron microscopy
TMN	Total mitochondrial number
pS65	Phospho-ubiquitin serine 65
ATP	Adenosine triphosphate
AF	Autofluorescence
ROS	Reactive oxygen species
RNS	Reactive nitrogen species
AMPK	5′-adenosine monophosphate-activated protein kinase
NaIO3	Sodium iodate
NAC	N-acetylcysteine

## Appendix A

Since mitophagy may be activated without PINK1/PARKIN, we attempted to test the roles of other mitophagy receptors. Recent studies have identified eight mechanistically different mitophagy receptors. One group contains a ubiquitin-binding domain which binds to ubiquitinated mitochondria [20], such as p62/SQSTM1, NBR1 (Neighbor of BRCA1 gene 1 protein), optineurin (OPTN) [67,68] and AMBRA1 (autophagy and beclin-1 regulator 1) [69,70]. The second group of mitophagy receptors [20] contains a transmembrane domain that can directly bind to the OMM and stimulate autophagosome formation via their LIR motifs, namely Bnip3 (BCL2 interacting protein 3) [71], Nix/Bnip3L (BCL2-like 3) [72], FUNDC1 (FUN14 domain containing 1) [73] and Bcl2L13 (BCL2-like 13) [74]. Their roles in the regulation of cell proliferation, cytosolic protein degradation and cell death have also been reported [75,76,77]. However, their importance in mitochondrial clearance is not clearly known. It has been reported that high-glucose-induced ROS generation inhibited mitophagy via ROS/PINK1/PARKIN signaling pathway in RPE cells, whereas overexpression of PINK1 or PARKIN could reverse this effect [78].

In conditions of oxidative stress and elevated mitochondrial damage, we anticipated that it would be possible to detect colocalization of AMBRA1, Bnip3 and FUNDC1 with a mitochondrial marker ATP synthase β in dKOs. The colocalization of mitochondria with respective mitophagy activated receptors would be one way of gaining insights into their role in our dKO animals. Although there was a ~3% increase in the total number of puncta, the colocalization of AMBRA1 with ATP synthase β was reduced by ~18% as compared to WT (Figure A1a). In the evaluation of the amount of mitophagy receptor Bnip3, the total number of puncta was reduced by ~13% with a ~27% reduction in colocalization as compared to WT (Figure A1b). Interestingly, FUNDC1 in dKO RPE showed a ~30% increase in total puncta along with a ~45% increase in colocalization. However, the differences between all these stainings were not statistically significant in comparison to WT (Figure A1c). Based on these results, these ubiquitin-independent selective autophagy receptors are not involved in the mitophagy initiation process in our dKO model.
