# Supplementary material for: Mitophagy in the Retinal Pigment Epithelium of Dry Age-Related Macular Degeneration Investigated in the NFE2L2/PGC-1α-/- Mouse Model
Source: Int J Mol Sci. 2020 Mar 13;21(6):1976. doi: 10.3390/ijms21061976 (PMC7139489; doi:10.3390/ijms21061976)
Supplement: Supplementary file 1 [file ijms-21-01976-s001.pdf]

Supplementary data

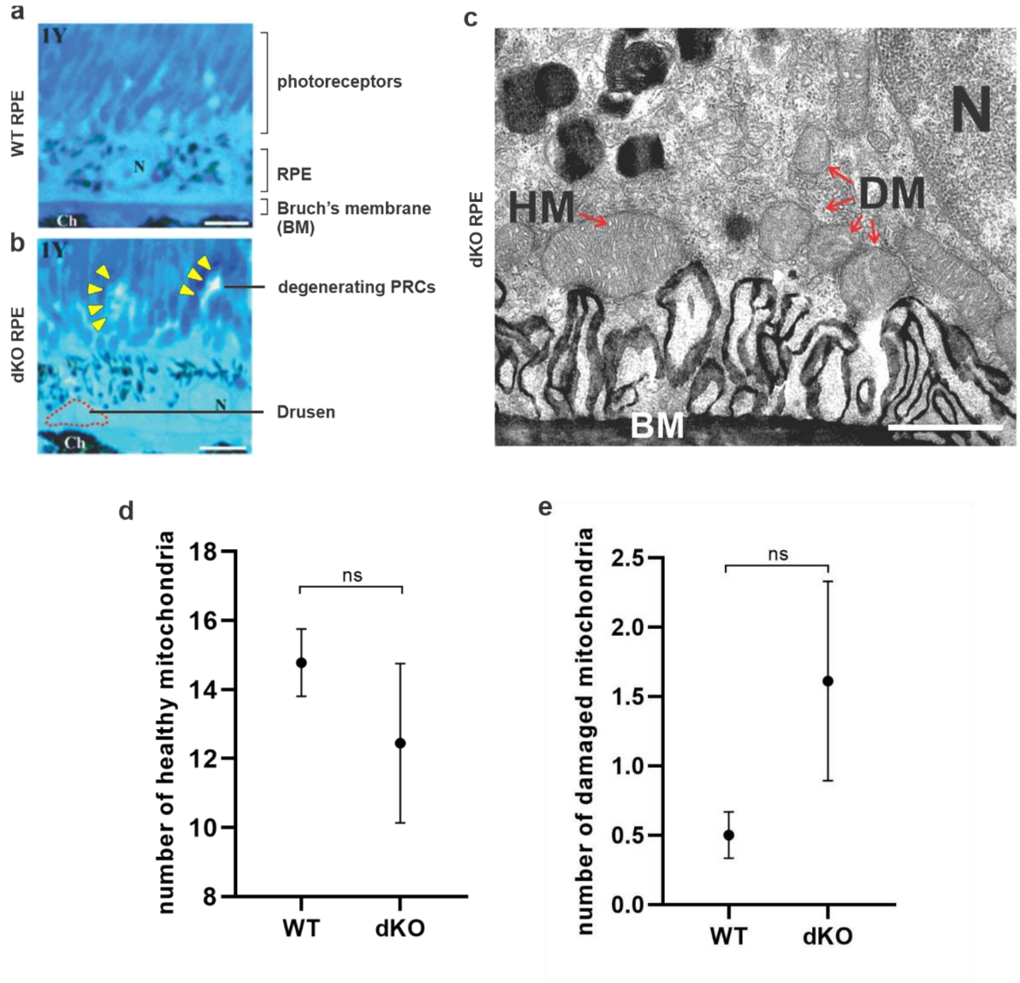

Supplementary figure 1. The toluidine blue stained sections from the posterior part of the eye demonstrate the layers of the retina and the morphological relationship of photoreceptor/RPE/Bruch`s membrane/ complex (**a**). The layers of sensory retina are clearly visible. *NFE2L2/PGC-1α* dKO shows positive signs of dry AMD including drusen-like deposits (red dotted lines) (**b**). The total numbers of healthy and damaged mitochondria were counted from 6 WT and 6 dKO using TEM imaging (**c**). The total numbers of healthy mitochondria (**d**) were reduced in dKO (p = 0.70); correspondingly, the numbers of damaged mitochondria (**e**) were increased in dKO (p = 0.19). Data are presented as mean ± SEM, Scale = 5 μm (**a**,**b**) and 1 μm (**c**). Healthy mitochondria (HM), damaged mitochondria (DM), Nuclei (N) and Bruch's membrane (BM). ns- non significance.

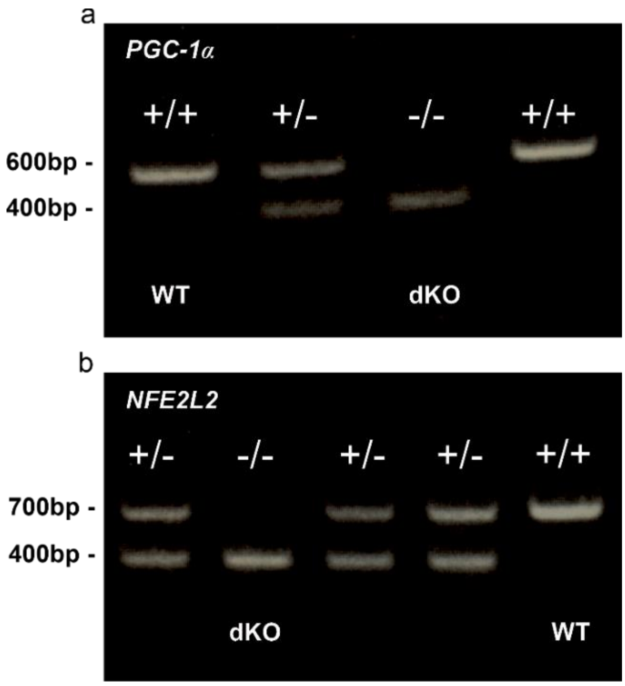

Supplementary figure 2. Genotyping of *NFE2L2* and *PGC-1α* double knockout mice (dKO) and wild type (WT).
